# Gamma Frequency and the Spatial Tuning of Primary Visual Cortex

**DOI:** 10.1371/journal.pone.0157374

**Published:** 2016-06-30

**Authors:** Sarah Gregory, Marco Fusca, Geraint Rees, D. Samuel Schwarzkopf, Gareth Barnes

**Affiliations:** 1 Wellcome Trust Centre for Neuroimaging, UCL, London, WC1N 3BG, United Kingdom; 2 Institute of Cognitive Neuroscience, UCL, London, WC1N 3AR, United Kingdom; 3 Center for Mind/Brain Sciences, University of Trento, Mattarello, Italy; 4 Experimental Psychology, UCL, London, WC1H 0AP, United Kingdom; Radboud University Nijmegen, NETHERLANDS

## Abstract

Visual stimulation produces oscillatory gamma responses in human primary visual cortex (V1) that also relate to visual perception. We have shown previously that peak gamma frequency positively correlates with central V1 cortical surface area. We hypothesized that people with larger V1 would have smaller receptive fields and that receptive field size, not V1 area, might explain this relationship. Here we set out to test this hypothesis directly by investigating the relationship between fMRI estimated population receptive field (pRF) size and gamma frequency in V1. We stimulated both the near-center and periphery of the visual field using both large and small stimuli in each location and replicated our previous finding of a positive correlation between V1 surface area and peak gamma frequency. Counter to our expectation, we found that between participants V1 size (and not PRF size) accounted for most of the variability in gamma frequency. Within-participants we found that gamma frequency increased, rather than decreased, with stimulus eccentricity directly contradicting our initial hypothesis.

## Introduction

The primary visual cortex (V1) is composed of columnar aggregations of neurons with similar tuning properties [1–3]. The size and width of these columns relates to the cortical surface area of V1 [4]. Predictably, there is substantial individual variability in V1 surface area which can greatly affect visual perception [5–7]. For example, those people with larger surface area are less susceptible to certain visual illusions as they fail to use broader visual contextual information compared to those with smaller V1 surface areas.

Visual stimulation produces oscillatory electrical activity in visually responsive neuronal populations that can be measured using magnetoencephalography (MEG). These neuronal dynamics are most evident in the gamma-band frequency of 30-80Hz and have been linked to perceptual and cognitive function [8]. In particular, there is a positive association between higher frequency within the gamma band and neuronal tuning and behavioral discrimination of stimulus orientation [9, 10]. Similarly, neurons focused in more homogeneous regions of the cortex demonstrate more uniform orientation preference and therefore, sharper tuning [11]. Furthermore, recent studies have demonstrated a close correlation between gamma peak frequency in the visual cortex and certain features of visual stimuli, such as contrast and velocity [12–15] and visual processing abilities [9, 16, 17].

In a recent study, we identified a positive correlation between retinotopically-determined surface area of central V1 and peak gamma frequency [18]. As there was no association with volume, we suggested that the higher peak gamma frequency was potentially due to the smaller receptive field sizes (greater cortical magnification, or greater local homogeneity in tuning properties) that one would expect in individuals with greater V1 surface area.

Here, we set out to test whether receptive field size could explain more of the variability in peak gamma frequency than V1 surface area. To do this we investigated the effect of both stimulus location and stimulus size (near-central or peripheral; large or small) in both hemispheres on peak gamma frequency. Firstly, we sought to replicate our previous finding [18] that V1 gamma frequency is associated with greater V1 surface area not only in a near-central location but also peripherally. Secondly, we measured population receptive field sizes (pRF) corresponding to the four different locations in V1 and related their sizes to peak gamma frequency. The pRF is an estimate of the receptive field size of the population of neurons in a particular region of cortex (assessed using functional magnetic imaging (fMRI) [19]). Smaller pRFs indicate that the neuronal population is more selective to visual space (hence the cortical magnification, or amount of cortex per degree of visual space, is also greater). Given this, we made the between participants prediction that gamma peak frequency would be higher for individuals with smaller pRFs; and the within participants prediction that we would expect lower gamma peak frequencies to be generated for peripheral (where pRF size is larger) rather than central stimuli.

## Materials and Methods

### Participants

10 healthy individuals with normal or corrected to normal vision and with no neurological history (mean 29.1 years (± 5.34), three female; 1 left-handed) participated in two experimental sessions, which took place on separate days. During the first session, retinotopic mapping was performed using fMRI. This session formed part of previous studies [20, 21]. We recruited as many participants as possible from these studies for the second session. During this session, participants were scanned with MEG to record V1 responses to a series of static grating stimuli which both varied in size and location. Participants gave written, informed consent. All procedures were in accordance with the Declaration of Helsinki and were approved by the University College London (UCL) Research Ethics Committee.

### Data collection

#### Magnetoencephalography (MEG)

**Fig 1:**
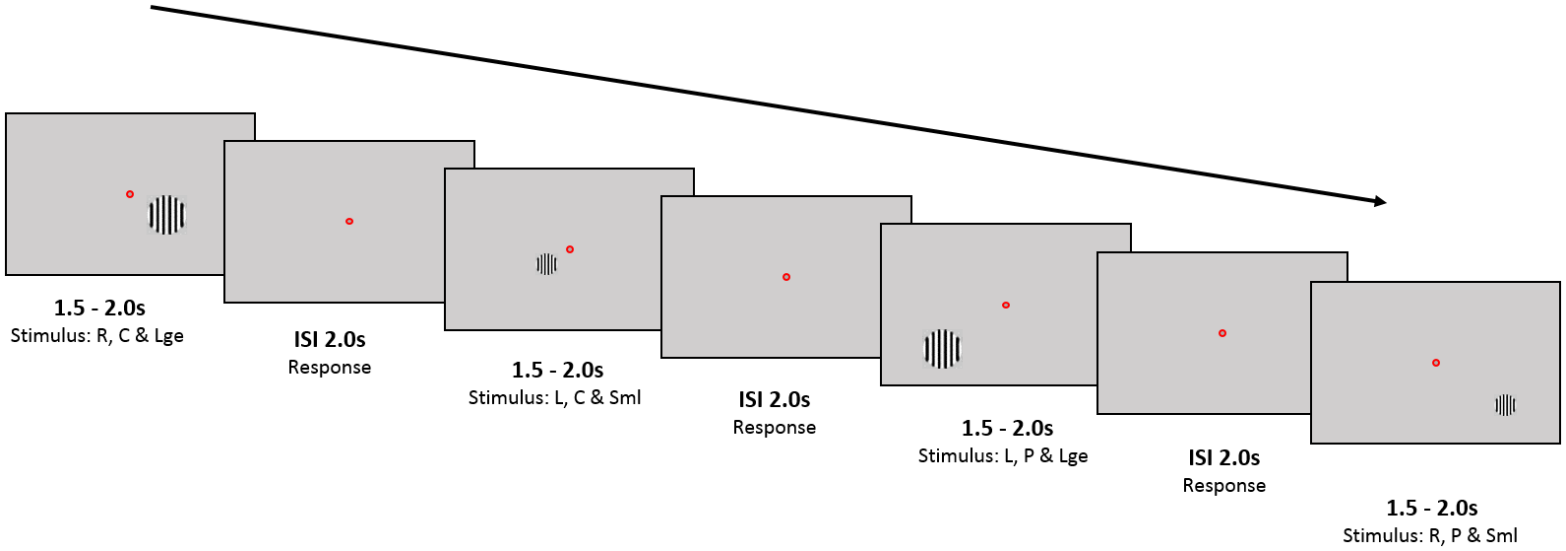
Schematic of MEG experiment: Example presentation of four stimuli Stimuli consisted of circular, static, high contrast, square-wave grating of 3 cycles/° spatial frequency on a mean luminance uniform grey background. In this figure, we show an example of a large, near-central stimulus presented to the right hemisphere (Block1); a small, near-central stimulus presented to the left visual hemifield (Block3); a large, peripheral stimulus presented to the left hemifield (Block5) and a small, peripheral stimulus presented to the right hemifield (Block7). These stimuli are spaced in time by an interstimulus interval of 1.5-2seconds. Participants were instructed to fixate on the red dot and respond whenever a stimulus disappeared. Abbreviations: R = Right; L = Left; C = Near-Central; P = Peripheral; Sml = Small; Lge = Large; ISI = Interstimulus interval.

Participants were seated in a MEG system and viewed visual stimuli on a projection screen placed in front of them. The size of the screen was 42x32cm and the participants were seated in the MEG scanner approximately 60cm from the screen. For every stimulus presented, participants were required to fixate on a small (0.2°) red dot in the center of the screen (Fig 1).

The stimuli consisted of a circular, static, high contrast, square-wave grating of 3 cycles/° spatial frequency on a mean luminance uniform grey background, presented either to the left or right hemifield, at two eccentricities (near-central and peripheral) and of two possible sizes (small: radius 0.8° and large: radius 1.4°) totaling eight sets of stimuli. We aimed to maintain the same area of active cortex (around one square centimeter) for eccentric and para-foveal stimuli. We also did not want the center of active cortex to change between small and large stimuli. We therefore used canonical (i.e. the same for all participants) M-scaling to estimate the mapping between visual space and V1[22]. Stimuli were presented on a diagonal axis crossing the lower visual field quadrants 45° obliquely distant centered respectively at ∼2.3°,2.52, 4.36, 4.5° from the center of gaze for near-central small, near-central large, peripheral small and peripheral large respectively. A desktop-mounted EyeLink II eyetracker (SR Research Ltd., Mississauga, ON, Canada), which samples eye position and pupil dilation at 250 Hz, was used to monitor eye movements.

Each run consisted of 160 trials. To minimize adaptation effects the contrast polarity was randomly reversed on half of the trials. The four stimuli (near-central small, near-central large, peripheral small, peripheral large) were shown either in the lower right visual field or in the lower left. These eight trial conditions were randomly interleaved and counterbalanced within a run. The interstimulus interval with the fixation point and grey background was set to two seconds. The stimuli were presented for a pseudo-random duration of between 1.5 and 2 seconds. Participants were instructed to respond with a button press whenever the stimulus disappeared. Participants performed four task runs; two with each hand, with half of the participants beginning with the left hand and the other half with the right hand (in the ABBA order).

Whole-head MEG recordings were made using a CTF axial gradiometer system with 275 channels, sampled at 600 Hz. To monitor participant head movement, three electrical coils were placed at fiducial locations. SPM12 (http://www.fil.ion.ucl.ac.uk/spm) with the DAISS (Data Analysis in Source Space) toolbox (https://code.google.com/p/spm-beamforming-toolbox/) running under MATLAB was used to analyze the MEG data. Recordings were divided into epochs from 1.5 s before stimulus onset until 1.5 s after stimulus onset (the earliest time for stimulus offset that preceded the participant’s behavioral response). One set of beamformer weights was calculated based on this (-1.5 to +1.5s) window in the 30-80Hz band for each of the four (left and right, near-center and peripheral) possible stimulus locations (small and large stimuli therefore shared the same beamformer weights). These weights were then used to make time series estimates at each source location. For each condition there were approximately 20 trials per run and per-trial power spectra were constructed from the periodograms of the Hanning windowed data in the post-stimulus (0 and 1.5s) and baseline periods (-1.5 to 0s). We then used a chi-squared test to construct an image of power change (in the 30-80Hz band) between post stimulus and baseline periods at each 5mm cubic voxel within an MNI masked occipital lobe. Based on this statistical image peak (using the original beamformer weights) we then calculated the peak power and peak frequency in the 30-80Hz band 0.5–1.5s post-stimulus (so as to avoid the evoked gamma component) for each hemisphere and stimulus. We also calculated one hundred bootstrapped resamples of a one-sampled t-test between power in stimulus vs. power in baseline conditions. Power was computed using a Hanning windowed periodogram estimated based on a fast Fourier transform of the data. The peak frequency was taken to be the mean peak frequency from across the bootstrap resamples.

#### Retinotopic mapping

Retinotopic mapping was performed as part of another study, for full details please see [20]. In brief, participants lay supine inside a Siemens 3T TIM-Trio scanner and viewed visual stimuli presented on a screen. Functional imaging data were acquired using a gradient echo planar imaging sequence (2.3 mm isotropic resolution, 30 transverse slices per volume, acquired in interleaved order and centered on the occipital cortex; matrix size: 96 × 96, slice acquisition time: 85 ms, TE: 37 ms, TR: 2.55 s); 148 volumes per mapping run and 124 volumes per hemodynamic response function (HRF) run. Only 20 channels of a 32-channel head coil were used due to impedance of participants' field of view. A double-echo FLASH sequence (short TE: 10 ms, long TE: 12.46 ms, 3 × 3 × 2 mm, 1 mm gap) was used to acquire B0 field maps to correct for field inhomogeneity and a T1-weighted structural image (1 mm isotropic resolution, 176 sagittal slices, matrix size 256 × 215, TE 2.97 ms, TR 1900 ms) was also collected. The full 32-channel head coil with a 3D modified driven equilibrium Fourier transform sequence (1 mm isotropic resolution, 176 sagittal partitions, matrix size 256 × 240, TE: 2.48 ms, TR: 7.92 ms, TI: 910 ms) was used as a basis for cortical reconstruction.

The fMRI experiments [20, 21] were divided into five functional runs: four for retinotopic mapping and one to estimate the HRF. During the mapping runs, participants fixated centrally on a dot in the center of the screen while a dynamic, high-contrast “ripple” pattern bar moved across the visual field, oriented either vertically or horizontally, moving in opposite directions, and interspersed with blank periods. This was repeated for 10 trials. Participants were required either to respond to a change in the color of the fixation dot [20] or were presented with a stream of differently-colored crosses and instructed to respond only to the red crosses irrespective of orientation [21]. All stimuli were generated in MATLAB R2012a (MathWorks) and displayed using the Psychtoolbox package (3.0.10).

Functional MR images were preprocessed using SPM8 (Wellcome Trust Centre for Neuroimaging, University College London). The T1 structural scan was segmented and underwent cortical reconstruction [23, 24] using Freesurfer (version 5.0.0, http://surfer.nmr.mgh.harvard.edu). Any further analyses were performed using software developed in-house based in MATLAB (http://dx.doi.org/10.6084/m9.figshare.1344765). We projected functional data to the cortical reconstruction by identifying the voxel within the functional images that corresponded to the median location between the pial and white matter surface for each vertex of the cortical mesh. For each participant, we used a forward mapping approach to estimate pRF parameters for each vertex: center position in visual space (x,y), size of the center (σ_1_) and surround (σ_2_) component, the amplitude ratio of center and surround (δ), and an overall scale factor (β) [19, 20, 25, 26]. The predicted neural response was then convolved with the participant’s HRF fitted based on data from the HRF run. pRF parameters were then fitted to the time series for each vertex using a coarse-to-fine fitting approach in which we first performed an extensive grid search on heavily smoothed data followed by an optimization procedure applied to unsmoothed data. The final parameter maps were smoothed (FWHM 5mm on the spherical model) to reduce high frequency variability in the parameter estimates.

We manually delineated retinotopic visual regions as part of the earlier studies [20, 21] using Freesurfer. We then extracted the vertex data from each region in each hemisphere. To quantify pRF size at the four visual field locations of our stimuli in the MEG experiment, we calculated the mean pRF size (full width half maximum of the difference-of-Gaussians pRF model) across vertices with pRFs in the visual field quadrant and within the eccentricity range of the stimulus in the MEG experiment. To measure the macroscopic surface area of V1, we summed surface area estimates of all V1 vertices in the lower visual field maps whose pRF locations fell between 2° and 7° eccentricity. This way we excluded edge artifacts that otherwise could have added spurious variability between participants.

## Results

**Fig 2:**
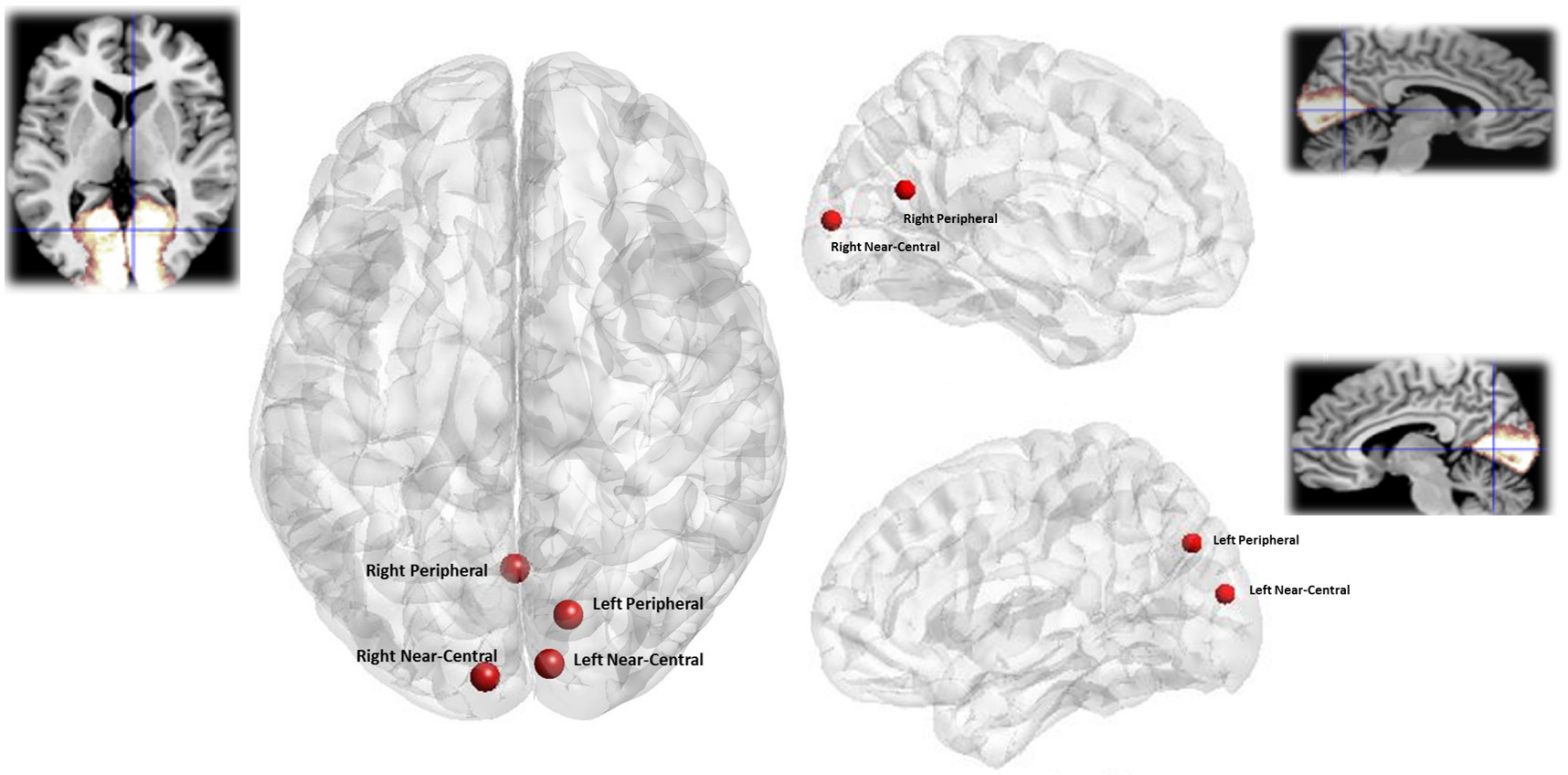
Location of visual stimuli responses. Visual cortex responses to each stimulus type averaged across all participants a) Axial view b) Left Hemisphere (sagittal view) c) Right Hemisphere (sagittal View). Insets show an anatomical mask of the primary visual cortex (BA17) derived from the Anatomy Toolbox http://www.fz-juelich.de/. All locations are based on MNI co-ordinates.

We treated data from the two hemispheres of each participant independently [25, 27, 28]. Average MNI co-ordinates for each location from which peak gamma frequency was extracted were: left near-central (x = -7.3, y = -92.6, z = 8.5) left peripheral (x = 0.5, y = -63.8, z = 20.7) right near-central (x = 8.8, y = -88.2, z = 15.7) right peripheral (x = 13.8, y = -74.9, z = 35.7) (Fig 2). Fig 2 shows the average location of the gamma power peaks in standard space. All peaks are above the calcarine consistent with lower-visual field stimulation and located in the contralateral hemisphere. The near-central peaks are close to the occipital pole as expected, and the power changes due to the more peripheral stimuli are more anterior, with the left-peripheral peak a little more superior than we would have expected.

Peak gamma frequency (for each hemisphere contralateral to that of the stimulus) and pRF size for each stimulus for each participant are detailed in Table 1.

**Table 1:** Visually induced peak gamma frequency and pRF size in V1 for each participant for each eccentricity and stimulus size. Participant data are treated independently for left and right hemisphere. V1 Cortical Surface Area (CSA) for each hemisphere is also included.

	Peak Gamma Frequency (Hz)				pRF FWHM (degs)				CSA (mm^2^)
	Near central		Peripheral		Near central		Peripheral		
	Small	Large	Small	Large	Small	Large	Small	Large	
**S1 Left**	53.05	58.52	60	60.86	1.959	2.038	2.584	2.55	615.779
**S1 Right**	55.04	60.88	58.57	53.57	2.414	2.353	2.134	2.21	336.087
**S2 Left**	58.3	50.56	67.85	67.16	1.993	1.999	1.816	1.808	671.240
**S2 Right**	54.52	53.12	55.9	62.91	2.42	2.435	2.35	2.377	598.496
**S3 Left**	63.86	64.2	61.03	57.39	1.861	1.985	1.928	1.853	835.664
**S3 Right**	64.66	63.69	68.12	63.5	2.3	2.219	2.271	2.179	621.871
**S4 Left**	54.44	53.95	63.37	48.2	2.387	2.27	3.009	2.815	487.154
**S4 Right**	46.11	46.57	55.13	52.27	2.783	2.85	3.159	3.076	277.116
**S5 Left**	55.82	58.31	64.34	59.46	2.026	2.022	2.215	2.181	470.330
**S5 Right**	64.43	65.3	55.81	63.69	1.936	1.85	1.685	1.719	534.935
**S6 Left**	49.35	53.98	60.6	53.6	2.179	2.159	2.058	2.103	426.407
**S6 Right**	50.76	47.05	57.04	66.05	2.293	2.013	1.991	1.938	440.312
**S7 Left**	46.13	46.3	67.81	70.41	1.888	1.917	1.937	1.941	466.267
**S7 Right**	62.98	67.23	50.9	64.14	1.626	1.733	2.034	1.907	531.209
**S8 Left**	46.02	41.15	52.2	63.03	2.021	2.019	2.416	2.367	561.819
**S8 Right**	43.5	43.61	49.79	46.41	1.789	1.852	1.935	2.026	469.439
**S9 Left**	57.94	62.09	65.15	57.68	1.831	1.881	2.015	2.032	624.599
**S9 Right**	55.65	57.35	64.88	59.56	1.796	1.763	1.8	1.825	678.816
**S10 Left**	48.11	46.88	67.03	69.77	2.088	2.169	2.085	2.121	783.356
**S10 Right**	64.01	67.31	52.29	64.04	2.005	2.043	2.055	2.049	554.710

We found (based on a repeated measures ANOVA) no significant effect of either stimulus size (F(1,17) = 1.294, p = 0.27), or eccentricity (F(1,17) = 0.923, p = 0.350) on source level gamma power.

**Fig 3:**
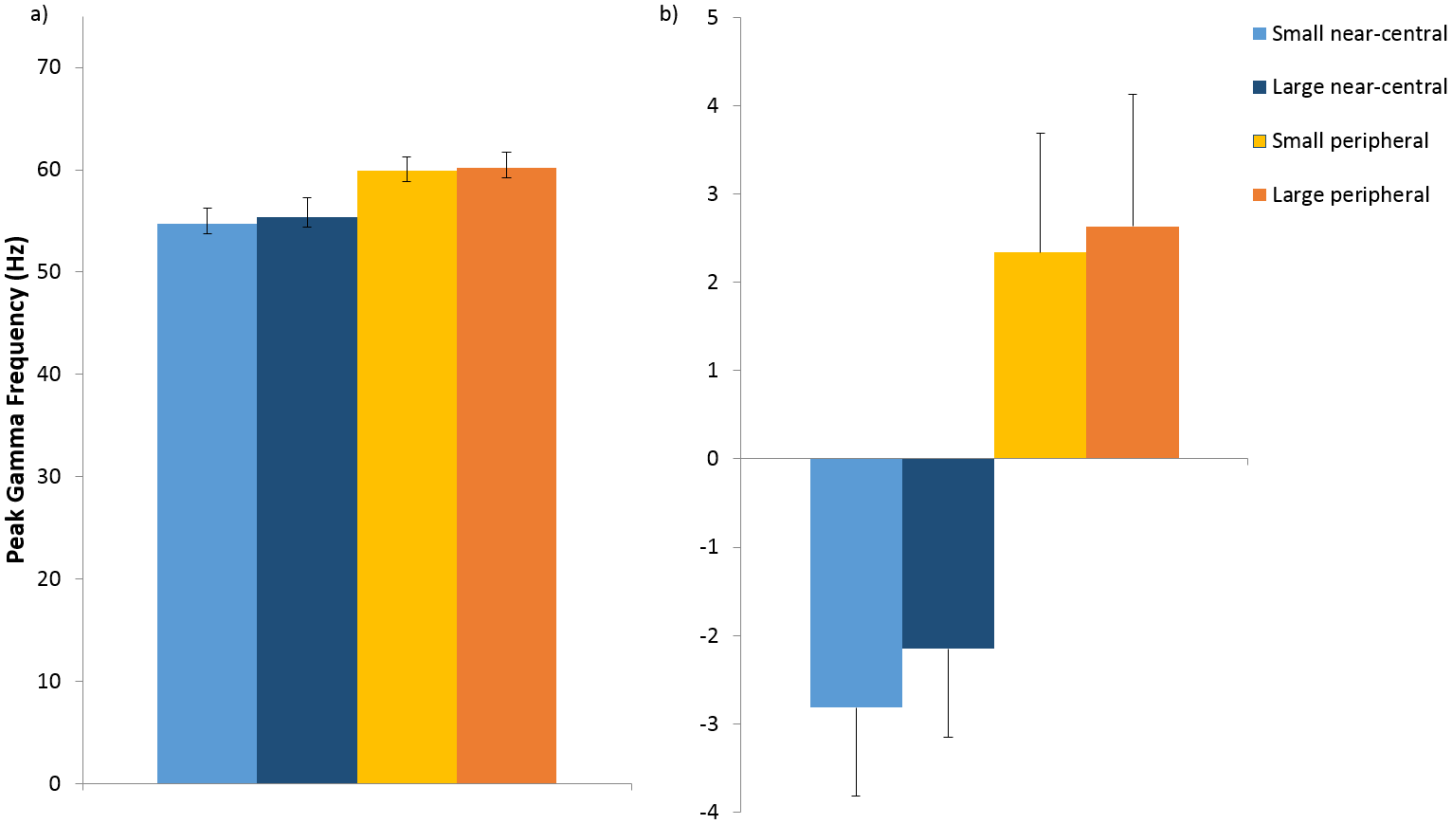
Peak Gamma Frequency induced by each visual stimulus. a) Average absolute peak gamma frequency for each stimulus (left and right hemisphere combined); b) Average within-participant mean-centered peak gamma frequency for each stimulus (left and right hemisphere combined). Error bars denote 1 standard error of the mean.

**Fig 4:**
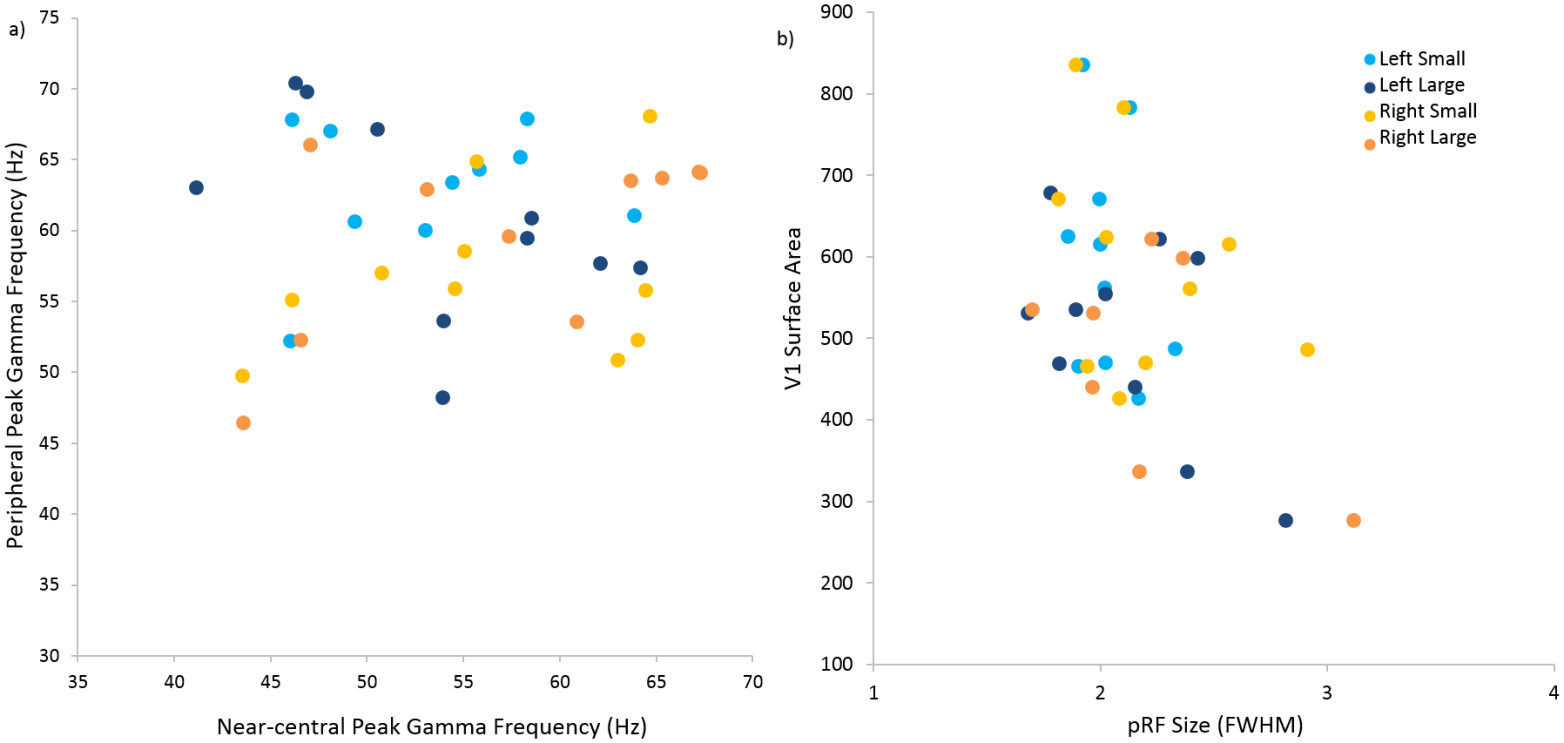
Peak Gamma Frequency, V1 surface area and Population Receptive Field. a) peak gamma frequency for near-central stimuli plotted against corresponding peak gamma frequency for peripheral stimuli for each hemisphere; b) pRF size plotted against V1 cortical surface area for each hemisphere. (Light blue circles = left small stimulus; dark blue circles = left large stimulus; light orange circles = right small stimulus; dark orange circles = right large stimulus).

A repeated measures ANOVA showed that there was an overall effect of stimulus eccentricity on peak gamma frequency (F(1,19) = 6.971, p = 0.017), but no main effect of stimulus size (F(1,19) = 0.307, p = 0.586) or interaction between stimulus size and eccentricity (F(1,19) = 0.035, p = 0.854). Average peak gamma frequency for the near-central stimulus was lower for both sizes (small: mean 54.73Hz, sd 6.87; large: mean 55.40Hz, sd 8.27) than that in the periphery (small: mean 59.89Hz, sd 6.05; large: mean 60.19Hz, sd 6.68) (Fig 3). We illustrate the relationship between peak gamma frequency for near-central stimuli and corresponding peak gamma frequency for peripheral stimuli for each hemisphere, in addition to the relationship between pRF size and V1 cortical surface area for each hemisphere (Fig 4).

**Fig 5:**
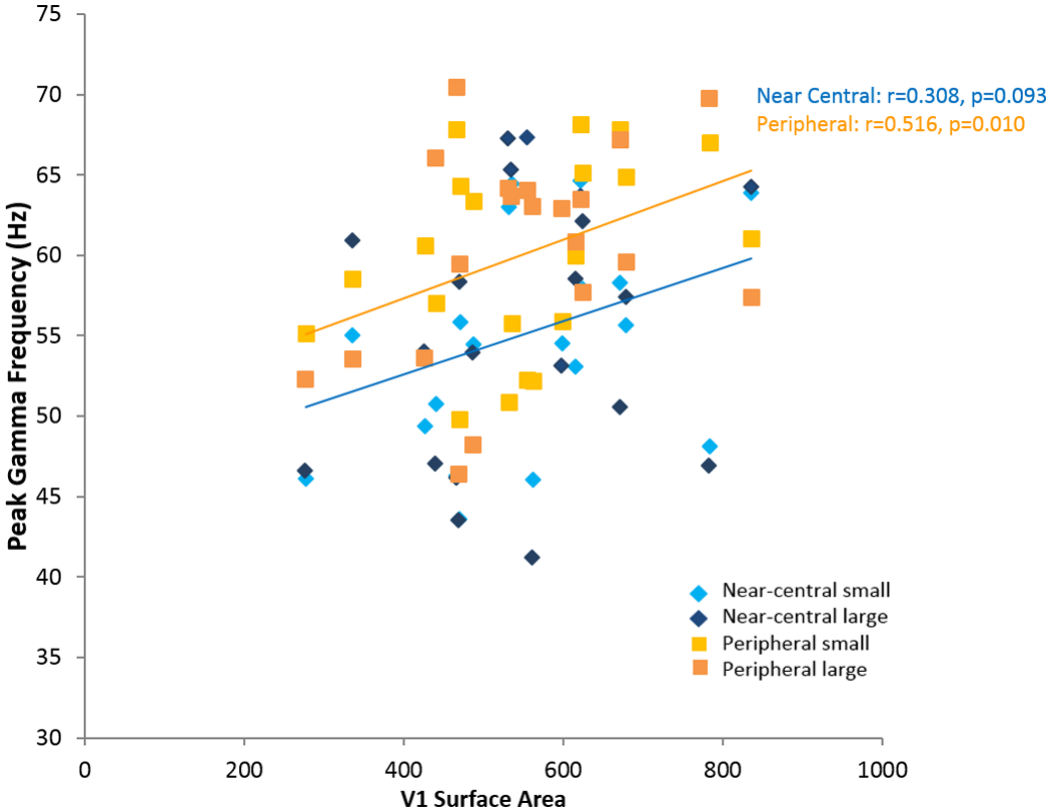
V1 Surface Area is positively correlated with peak gamma frequency Peak gamma frequency plotted against cortical surface area of V1 (right and left hemisphere). Linear regressions are shown for a) near-central stimuli (blue line) and b) peripheral stimuli (orange line). Each participant is represented by four points, for each hemisphere. (Light blue diamonds = near-central small stimulus; dark blue diamonds = near-central large stimulus; light orange squares = peripheral small stimulus; dark orange squares = peripheral large stimulus).

As there was no significant effect of stimulus size on peak gamma frequency, we averaged measurements for small and large stimuli for each eccentricity for the remaining analyses. We confirmed our previous finding that V1 surface area correlated positively with peak gamma for all combined stimuli (Spearman’s rho = 0.380, p = 0.008, one-tailed) (Fig 5).

Average pRF size was predictably larger for peripheral stimuli (mean 2.164°, sd 0.358°) than that of near-central stimuli (mean 2.079°, sd 0.267°). Average pRF size for each hemisphere was also negatively, but not significantly, correlated with our measure of cortical surface area (left: rho = -0.37, p = 0.147; right: rho = -0.43, p = 0.107, one-tailed) (Fig 4).

Our initial hypothesis had been that gamma frequency should increase with decreased pRF size, but the higher gamma frequency observed for more peripheral stimuli (with larger pRF size) within participants directly contradicted this (Fig 3). There was a trend towards a relationship between pRF size and gamma frequency between participants (rho = -0.241, p = 0.067, one-tailed) (Figure C in S1 File), a stepwise regression confirmed that this was due to shared variance with cortical surface area (R^2^ = 0.098, F(1,39) = 5.529, p = 0.027; V1 surface area: β = 0.349, p = 0.027; pRF size: β = -0.083, p = 0.626).

We then investigated whether the gamma frequency differences between near-central and peripheral stimuli within each individual could be predicted by the respective differences in the pRF sizes associated with those stimuli. We found no significant relationship (Spearman’s rho = 0.036, p = 0.440 one-tailed) between the difference in peak gamma frequency (near-central—peripheral) and the difference in pRF size (near-central—peripheral) for each participant.

## Discussion

We replicated our previous findings [18] by demonstrating a positive correlation between V1 cortical surface area and peak gamma frequency in particular for peripheral locations. There was, however, no evidence to support our prediction that higher peak gamma frequencies should be associated with smaller pRF size; as both within and between participants, the more eccentric stimuli (which should stimulate locations with larger pRFs) gave rise to increased gamma frequency. Finally, consistent with previous reports [29], we found that stimulus size did not influence gamma frequency.

We demonstrated that across participants, larger V1 area is robustly associated with higher peak gamma frequency. While we have previously demonstrated this relationship for stimuli presented near the central visual field [18], here, we have additionally shown that this is also the case for more peripheral stimuli and as such have demonstrated the robustness of the relationship between V1 surface area and peak gamma frequency. When analyzed separately the relationship between cortical area and gamma frequency remained significant for the peripheral stimulus ((r = 0.516, p = 0.01, one-tailed)) however the near-central stimulus (most similar to that used in the Schwarzkopf et al. study) did not (r = 0.308, p = 0.093, one-tailed). We attribute this to the smaller number of participants in this study and the additional experimental variance arising from having two stimulus size conditions at each eccentricity. Perry et al. have previously found no significant correlation between gamma frequency and the surface area of the entirety of V1 despite substantial inter-individual variability [30]. This may be in part due to methodological differences: Perry et al. used an automated probabilistic anatomical estimation of the whole of V1 based on structural MRI images [31], while we used retinotopic mapping to measure the visual angle sampled by the functional task only, that is, the central part of the visual field [18]. In previous work, we and others have shown a clear dissociation between these measures suggesting that the proportion of V1 cortex that is devoted to the fovea is not constant across individuals [27, 32, 33].

In this study we constructed separate beamformer weights for the near and peripheral stimuli. In order to verify that the observed frequency difference was not due to some source localization confound we performed the same analyses but used a single set of weights based on all conditions within a hemisphere. We found the same significant increase in frequency for near v.s. peripheral stimuli (F(1,19) = 7.788, p = 0.012), but no main effect of stimulus size (F(1,19) = 0.2119, p = 0.162) or interaction between stimulus size and eccentricity (F(1,19) = 0.035, p = 0.854). Average peak gamma frequency for the near-central stimulus was lower for both sizes (small: mean 54.55Hz, sd 5.42; large: mean 57.91Hz, sd 5.55) than that in the periphery (small: mean 56.77Hz, sd 7.69; large: mean 59.04Hz, sd 6.01) (Figure A in S1 File) and the positive relationship between cortical area and frequency was preserved (rho = 0.351 p = 0.013, one-tailed) (Figure B in S1 File).

Our initial hypothesis was that smaller receptive field sizes would give rise to an increase in gamma peak frequency. We based this prediction on recent evidence demonstrating a positive association between higher frequency within the gamma band and orientation discrimination [9, 10] and that in turn neuronal assemblies that are located in areas of greater homogeneity have sharper tuning properties [11]. Inversely related to cortical surface area [25, 34], pRF measurements provide a statistical summary of neuronal tuning properties for every voxel within a stimulated region of the visual cortex [19]. Importantly, the suggestion that the smaller receptive field sizes give rise to higher gamma frequencies was directly contradicted by the within participant effects. Principally, we found that peak gamma frequency was actually higher for peripheral (large pRF size) than centrally (small pRF) located stimuli; this difference in gamma frequency was not related to the difference in pRF size at the level of individual participants. This finding is also inconsistent with previous reports [29]. This finding may in part be due to the use of different stimuli although it is unclear why Van Pelt’s stimuli should produce such a different pattern of results [29]. Whilst their annular stimuli are likely to produce higher gamma power than our square way grating stimuli [35], it is less obvious why gamma frequency should differ and why this should interact differently with eccentricity. One important point is that in this study we aimed to keep the stimulated area of active cortex constant as eccentricity varied and we did this by making the assumption that all participants had the same generic cortical magnification factor. It is possible therefore that some of the differences in gamma frequency observed are due to differences in the effective stimulus sizes on the cortex. For example, the eccentric stimuli may give rise to a higher frequency as there is less cortical area (less lateral inhibition) active as compared to the more central stimuli. However, although in this study there was some indication that larger stimuli give rise to higher frequency oscillations (see Fig 3) this effect was not significant (using either individual weights (F(1,19) = 0.307, p = 0.586), or common weights (F(1,19) = 0.2119, p = 0.162).

Previous work has suggested that local concentrations of the neurotransmitter GABA in visual cortices may be related to higher frequency gamma oscillations [17]. Greater GABA concentration putatively leads to increased inhibition in visual cortex, sharpening orientation tuning and the frequency of gamma oscillations [9, 17]. Despite some evidence that there is a higher density of GABA receptors in V1 [36], there has been considerable controversy as to the association between GABA concentration and gamma frequency [37, 38]. However, a recent study has demonstrated a direct relationship between the density of GABA_a_ receptors and gamma frequency in human primary visual cortex [39]. This study used PET to quantify levels of GABA_a_ receptor density and MEG to measure gamma oscillations in the primary visual cortex following visual task stimulation and has shown that that GABA_a_ receptor density correlates positively with the frequency of gamma oscillations. Since our results suggest that gamma frequency is higher in individuals with larger V1 area, it is possible that a larger V1 is therefore also associated with greater GABA concentrations [18]. This is further supported by evidence that there is a higher density of GABA receptors in V1 [36]. Recent research, for example, has demonstrated that the cortical surface area of the V1 cortex is directly related to higher GABA concentration [40] and may thus ultimately be linked to increased frequency of gamma oscillations.

Currently, we can only remove spatial tuning as one of the main factors explaining gamma frequency variation within an individual. Indeed, perhaps given the large amount of literature given to modelling the behavior of interacting pools of neurons (see for example [4, 17, 41–43]), future work needs to address more directly the relationship between location-specific changes in peak gamma frequency and underlying neuronal architecture.

## Supporting Information

S1 File **A: Peak Gamma Frequency induced by each visual stimulus using combined beamformer weights** a) Average absolute peak gamma frequency for each stimulus (left and right hemisphere combined); b) Average within-participant mean-centered peak gamma frequency for each stimulus (left and right hemisphere combined). Error bars denote 1 standard error of the mean. **B: V1 Surface Area is positively correlated with peak gamma frequency** Peak gamma frequency plotted against cortical surface area of V1 (right and left hemisphere). Linear regressions are shown for near-central stimuli (blue circles) and peripheral stimuli (orange circles). Each participant is represented by four points, for each hemisphere. **C: Peak gamma frequency plotted against pRF size (right and left hemisphere)** Each participant is represented by two points, one for each hemisphere. (Light blue diamonds = near-central small stimulus; dark blue diamonds = near-central large stimulus; light orange squares = peripheral small stimulus; dark orange squares = peripheral large stimulus). Linear regressions are shown for a) central stimuli (blue line) and b) peripheral stimuli (orange line); note however that this covariation seems to be driven primarily by the covariation of V1 area with both gamma frequency and PRF size.
(DOCX)
