# Supplementary material for: Gamma Frequency and the Spatial Tuning of Primary Visual Cortex
Source: PLoS One. 2016 Jun 30;11(6):e0157374. doi: 10.1371/journal.pone.0157374 (PMC4928794; doi:10.1371/journal.pone.0157374)

**Supplementary Information**

**S1 Figure**

**A: Peak Gamma Frequency induced by each visual stimulus using combined beamformer weights.** a) Average absolute peak gamma frequency for each stimulus (left and right hemisphere combined); b) Average within-participant mean-centered peak gamma frequency for each stimulus (left and right hemisphere combined). Error bars denote 1 standard error of the mean.


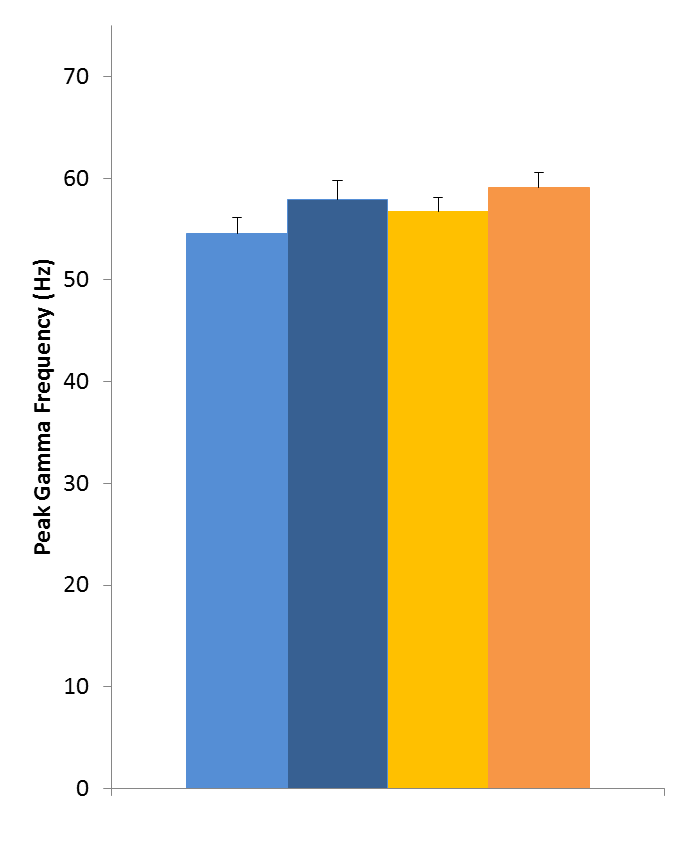

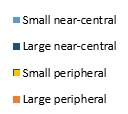


**B: V1 Surface Area is positively correlated with peak gamma frequency** Peak gamma frequency plotted against cortical surface area of V1 (right and left hemisphere). Linear regressions are shown for near-central stimuli (blue circles) and peripheral stimuli (orange circles). Each participant is represented by four points, for each hemisphere.

**C: Peak gamma frequency plotted against pRF size (right and left hemisphere).** Each participant is represented by two points, one for each hemisphere. (Light blue diamonds = near-central small stimulus; dark blue diamonds = near-central large stimulus; light orange squares = peripheral small stimulus; dark orange squares = peripheral large stimulus). Linear regressions are shown for a) central stimuli (blue line) and b) peripheral stimuli (orange line); note however that this covariation seems to be driven primarily by the covariation of V1 area with both gamma frequency and PRF size.


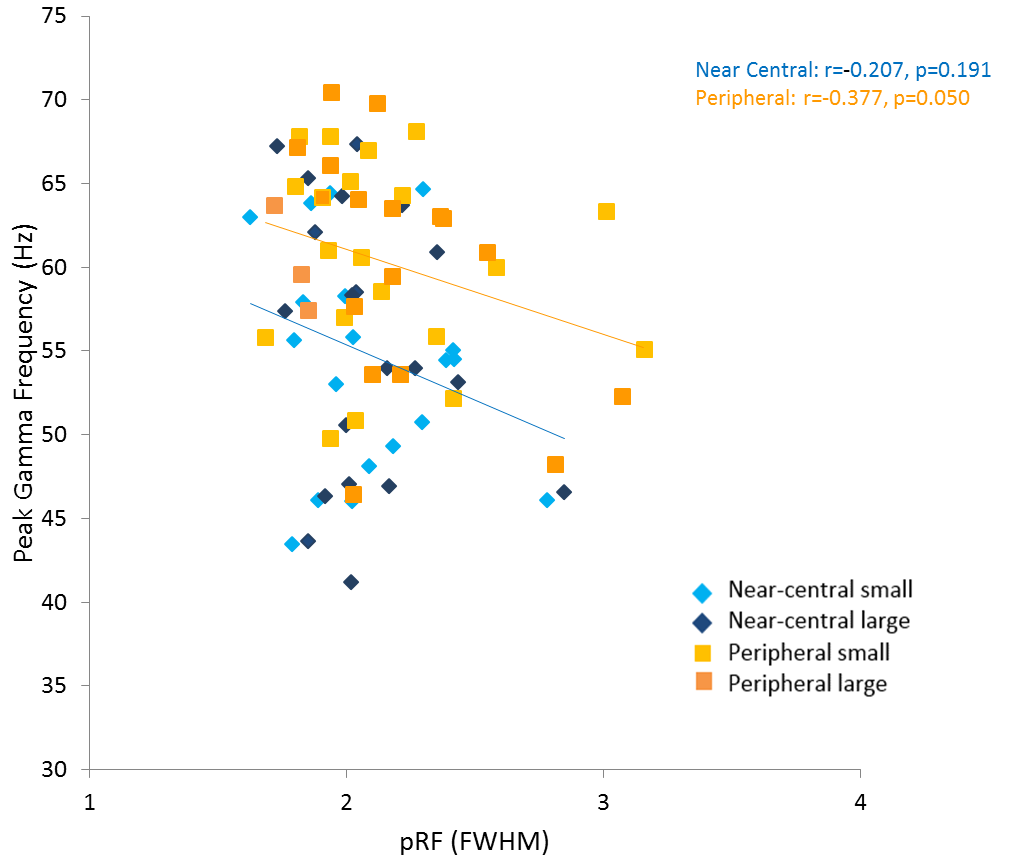

Supplement: S1 File — A: Peak Gamma Frequency induced by each visual stimulus using combined beamformer weights a) Average absolute peak gamma frequency for each stimulus (left and right hemisphere combined); b) Average within-participant mean-centered peak gamma frequency for each stimulus (left and right hemisphere combined). Error bars denote 1 standard error of the mean. B: V1 Surface Area is positively correlated with peak gamma frequency Peak gamma frequency plotted against cortical surface area of V1 (right and left hemisphere). Linear regressions are shown for near-central stimuli (blue circles) and peripheral stimuli (orange circles). Each participant is represented by four points, for each hemisphere. C: Peak gamma frequency plotted against pRF size (right and left hemisphere) Each participant is represented by two points, one for each hemisphere. (Light blue diamonds = near-central small stimulus; dark blue diamonds = near-central large stimulus; light orange squares = peripheral small stimulus; dark orange squares = peripheral large stimulus). Linear regressions are shown for a) central stimuli (blue line) and b) peripheral stimuli (orange line); note however that this covariation seems to be driven primarily by the covariation of V1 area with both gamma frequency and PRF size. (DOCX) [file pone.0157374.s001.docx]
